# The combined effects of filter-feeding bivalves (*Cristaria plicata*) and submerged macrophytes (*Hydrilla verticillate*) on phytoplankton assemblages in nutrient-enriched freshwater mesocosms

**DOI:** 10.3389/fpls.2023.1069593

**Published:** 2023-01-23

**Authors:** Xue Du, Dan Song, Huibo Wang, Jingshuang Yang, Hui Liu, Tangbin Huo

**Affiliations:** ^1^ Key Laboratory of Aquatic Organism Protection and Ecological Restoration in Cold Waters, Heilongjiang River Fisheries Research Institute, Chinese Academy of Fishery Sciences, Harbin, China; ^2^ Heilongjiang River Basin Fisheries Ecology Observation and Research Station of Heilongjiang Province, Harbin, China; ^3^ Jilin Chagan Lake National Nature Reserve Administration, Songyuan, China

**Keywords:** biomanipulation, control of cyanobacteria, eutrophication, filer-feeding bivalves, submerged macrophytes

## Abstract

Freshwater ecosystems are threatened by eutrophication, which causes persistent and harmful algal blooms. Filter-feeding bivalve mollusks and submerged macrophytes (SMs) alleviate the eutrophication effects by inhibiting phytoplankton biomass blooms. However, very little is known about whether and how the combined manipulation of filter-feeding bivalves and SMs control eutrophication and influence phytoplankton assemblages. Here, we performed a nutrient-enriched freshwater mesocosm experiment to assess the combined effects of the filter-feeding bivalve *Cristaria plicata*, a cockscomb pearl mussel, and the macrophyte *Hydrilla verticillate* on the biomass and composition of phytoplankton assemblages. We found that addition of *C. plicata* and *H. verticillate* decreased the water nutrient concentrations and suppressed overall phytoplankton biomass. Further, distinct differences in taxa between restoration and control treatments were observed and noticeably competitive exclusion of cyanobacteria in the restoration treatments occurred. An antagonistic interaction between filter-feeding bivalves and SMs was only detected for total cyanobacteria biomass demonstrating that a larger magnitude of SM restoration may override the effect of filter-feeding bivalves. Our results suggest that manipulation, through the addition of bivalves as grazers, associated with the restoration of SMs, is an efficient approach for reducing cyanobacterial blooms and alleviating eutrophication.

## 1 Introduction

Eutrophication of freshwater ecosystems, driven primarily by over enrichment of nitrogen (N) and phosphorus (P) (Carpenter et al., 1998), is a serious threat to water quality, biodiversity and other key ecosystem functions (Smith et al., 2006; Cook et al., 2018; Liu et al., 2021). Nutrient enrichment promotes the appearance and persistence of harmful algal blooms (Heisler et al., 2008; Conley et al., 2009) and the decline of submerged macrophytes (SMs) (Zhang et al., 2017), altering the food web structure (Fujibayashi et al., 2018; Briland et al., 2020). Occurrences of eutrophication are expected to increase with climate and land-use changes (Jeppesen et al., 2010; Bergström and Karlsson, 2019; Le Moal et al., 2019), inducing regime switches from a macrophyte‐dominated clear state to phytoplankton‐dominated turbid state (Jeppesen et al., 2007a). Considering that human activity is the primary cause of the eutrophication, it is crucial to reduce anthropogenic contributions to aquatic ecosystems and to find effective approaches to control cyanobacterial blooms that usually dominate eutrophic waterbodies.

The restoration of SMs is considered a crucial measure for the rehabilitation of shallow eutrophic lakes (Liu et al., 2020; Li et al., 2021a), as SMs display certain functional traits, that they use to stabilize the clear-water state (Puijalon et al., 2011; Su et al., 2019; Rao et al., 2020). For example, SMs could suppress algal growth by competing for light and nutrients (Lürling et al., 2006), producing algae-inhibiting allelochemicals to interfere the photosynthetic activities (Zhu et al., 2010) and change other physiological and biochemical processes (Zhu et al., 2021; He et al., 2023), and providing grazing zooplankton with a daytime refuge against fish predation (Burks et al., 2001). In addition, SMs can facilitate nutrient uptake from the water column and sediment (Sand-Jensen and Borum, 1991) and reduce sediment resuspension (Horppila and Nurminen, 2003). Earlier studies involving small-scale experiments (Barrow et al., 2019; Amorim and Moura, 2020) and natural aquatic ecosystems (Chao et al., 2022; Peng et al., 2022) have repeatedly reported that the restoration of SMs decreases the phytoplankton abundance and increases water clarity. Thus, usage of SMs is a prospective tool for the elimination of algal blooms (Jeppesen et al., 2007b). The submerged macrophyte restoration is, therefore, expected to prevent or mitigate the expansion of cyanobacterial blooms.

Another restoration technique to improve water quality is the biomanipulation of filter-feeding freshwater animals, such as mussels; however, its effectiveness remains debatable. For example, grazing studies involving filter-feeding mussels, such as zebra mussels and triangle sail mussels, in Europe and China demonstrated that they can efficiently consume pelagic algae and detritus (e.g. MacIsaac et al., 1992; Gao et al., 2017). Further, mussels, as grazers, can reduce or even prevent algal blooms (Gulati et al., 2008). Furthermore, Wu and Culver (1991) found abundant zebra mussels in Lake Erie, and noticed that filter-feeding *Daphnia* were able to reduce edible algal density and enhance water transparency. Interestingly, some mussels display food selectivity and avoid consuming cyanobacteria resulting in dominance of cyanobacteria over other forms (Hwang et al., 2004; Colvin et al., 2015). Contrary to Hwang et al. (2004) and Baker et al. (1998); Colvin et al. (2015) reported that the invasion of zebra mussels led to a decline in *Microcystis* biomass in the Hudson River.

Numerous combined technologies for controlling lake eutrophication have been developed, demonstrating that the combined effect of the two technologies was better than the technology alone. For example, the combination of large herbivorous zooplankton and submerged macrophytes proved to be more efficient at controlling the biomass of cyanobacteria (Amorim and Moura, 2020). In addition, the successful restoration of submersed macrophytes improved the water quality in a eutrophic lake after the removal of common carp (Knopik and Newman, 2018). Despite recent advances on biological restoration methods related to eutrophication, little is known about whether and how the combined manipulation of filter-feeding bivalves and SMs control eutrophication and influence phytoplankton assemblages. Given the complexities of climatically, thermally, ecologically, and hydrologically induced change in natural lakes (Richardson et al., 2019), mesocosm studies have been heralded as a useful means to investigate the effects of multiple factors under manipulated or controlled environmental conditions while supporting realistic levels of biocomplexity (Stewart et al., 2013; Fordham, 2015).

In this study, we designed a 32-day nutrient-enriched freshwater mesocosm experiment to explore the potential interactions between the filter-feeding bivalves and SMs and their impact on the biomass and composition of phytoplankton assemblages. We reasoned that categorizing cyanobacteria based on the adaptations to avoid predation (e.g. colonial and filamentous cyanobacteria) may lead to greater insights into the combined effects of filter-feeding bivalves and SMs on the restoration of eutrophic water bodies. We hypothesized that: (i) biomanipulation *via* addition of filter-feeding bivalves and restoration of submerged macrophyte, under nutrient enrichment, will likely affect phytoplankton assemblages and control the growth of cyanobacteria; (ii) the interactive effects are likely to be superior to either alone for controlling eutrophication.

## 2 Material and methods

### 2.1 Study site and experimental design

**Figure 1:**
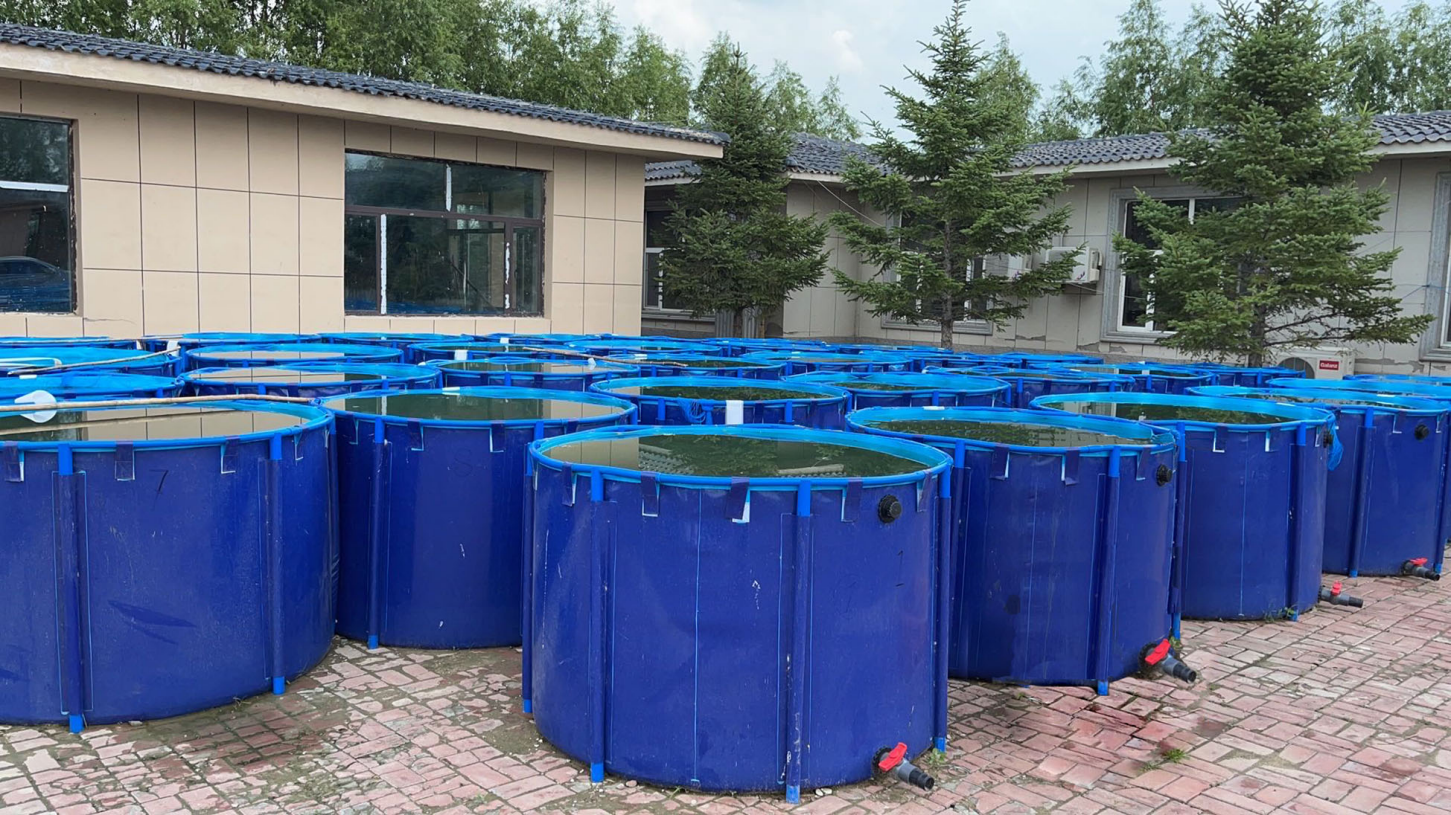
Experimental mesocosms used in our study at the Chagan Lake Observation and Research Station.

The outdoor mesocosm experiment was conducted between 25 June and 27 July 2021 in 16 cylindrical polyethylene mesocosms on land – at the Chagan Lake Observation and Research Station near Chagan Lake (45.25°N, 124.28°E). The mesocosms had a diameter of 1 m and a constant water depth of 1.2 m; they contained 0.2 m sediment and 780 L of unfiltered water collected from Chagan Lake (**Figure 1**). Chagan Lake is a shallow eutrophic freshwater lake (mean depth: 2.5 m) in a catchment area dominant by agricultural lands and grasslands and has relatively high allochthonous inputs of nutrients (especially nitrogen and phosphorus) through precipitation and surface run-off (Liu et al., 2019; Du et al., 2022). Nitrogen and phosphorus, as dissolved mixtures of sodium nitrate (NaNO_3_) and potassium dihydrogen phosphate (KH_2_PO_4_), respectively, were added daily to each mesocosm to equate to a nutrient load of 36 μg/L and 5 μg/L, which adhered to the Redfield ratio (Redfield, 1958). The walls of the mesocosms were scrubbed daily to prevent periphyton growth. During the experiment period, evaporation losses from the mesocosms were replaced with unfiltered lake water when not compensated for by rainfall.

The experiment had a factorial design (2 x 2) to evaluate of the effects bivalves, macrophytes, and their interaction, on water nutrient concentrations and phytoplankton assemblages in mesocosms. *Cristaria plicata* was chosen as the filter-feeding bivalve in our mesocosms as it is an excellent cleaner of suspended particles (Yu et al., 2020), and *Hydrilla verticillate*, was used as the macrophyte owing to its allelopathic effects on phytoplankton (Gao et al., 2015) and nutrient removal capability (Li et al., 2021b). Three treatments (bivalve: *C. plicata* alone; macrophyte: *H. verticillate* alone; bivalve + macrophyte: *C. plicata* and *H. verticillate* together) and a control (both species absent), each one with four replicates, were randomly assigned to the mesocosms and all received a common nutrient loading over the entire experiment. The bivalve, macrophyte, and bivalve + macrophyte treatments have been proposed as strategies for mitigating eutrophication for many temperate shallow lakes, and consequently, served as the restoration treatments (Søndergaard et al., 2007; Zhang et al., 2014). We added two *C. plicata* with a biomass of 413.8 ± 13.6 standard error (*S.E.*) g/m^2^ to the bivalve treatment mesocosms. *C. plicata* were hung with string bags, 30 cm above the sediment surface. Individual *H. verticillate* samples were purchased from a commercial nursery. At the beginning of the experiment, the average stem length of *H. verticillate* was 34 ± 0.8 *S.E.* cm and they were bundled together in groups of five to eight and weighted down in the sediment to encourage root growth. The total wet weight of macrophytes within each mesocosm was 650 ± 10.3 *S.E.* g L^-1^.

### 2.2 Sample collection and analysis

Samples of water nutrients and chlorophyll *a* concentrations were collected at the beginning of the experiment (day 0) and on day 4, 8, 12, 16, 20, 24, 28, and 32. The water samples were collected with a tube sampler at two different depths (surface and 5 cm above the sediment), from which subsamples were taken for water nutrients and phytoplankton analysis. We determined concentrations of total phosphorus (TP), phosphate (PO_4_-P), total nitrogen (TN), ammonia nitrogen (NH_4_-N), nitrate nitrogen (NO_3_-N) and nitrite nitrogen (NO_2_-N) using standard methods (American Public Health Association, 1992). Chlorophyll *a* (as a proxy of total phytoplankton biomass) concentrations determined spectrophotometrically from matter retained on Whatman GF/C glass microfiber filters after cold ethanol extraction in darkness (Jespersen and Christoffersen, 1987).

To characterize the phytoplankton assemblage composition, we collected phytoplankton from all enclosures. Phytoplankton sampling was done at the beginning (day 0) and at the end of the experiment (day 32). A subsample of the mixed tube sample water was immediately fixed with Lugol’s solution. All samples were analyzed using a Sedgewick-Rafter counting chamber and an inverted microscope (RVL-100-G, ECHO, San Diego, California, USA). At least 500 natural units were enumerated and identified to the genus level (Hu and Wei, 2006). Cell volumes of each phytoplankton taxa were calculated after approximation to the nearest geometric standard solid (Hillebrand et al., 1999). The biomass estimates were calculated, assuming that the density of the organisms equals that of water (1 mm^3^ L^-1^ = 1 mg L^-1^) (Wetzel and Likens, 2000). As chlorophyll *a* (μg/L) measured using the spectrophotometric method and total phytoplankton biomass (mm^3^/L) estimated from microscope counts and measurements were positively correlated (*R^2^* = 0.82, *p* < 0.001), we used the latter measurement to estimate the biomass of cyanobacteria genera. In the case of cyanobacteria, species were classified into colonies and filaments based on their life form.

### 2.3 Statistical analyses

Prior to analyses, water nutrient and chlorophyll *a* concentration data were natural logarithm-transformed to meet the assumptions of normality and homoscedasticity when necessary. Principal response curve (PRC) method was used to evaluate the time‐dependent influence of the bivalve (*C. plicata*), submerged macrophyte (*H. verticillate*) and their potential interactions on key water nutrient concentrations in response to nutrient enrichment. The PRC method is a special case of partial redundancy analysis (RDA) and requires repeated observations from multiple time periods in order to represent the deviation in the treatments from the controls over time (Van den Brink and Braak, 1999; Oksanen et al., 2020). The statistical significance of the PRC models was tested using the Monte Carlo permutation test (Van den Brink and Braak, 1999). The PRC analyses displayed an affinity for the different water nutrient (response) variables with the trajectory by giving each variable a quantitative score. In our study, higher scores of water nutrient variables in a restoration treatment group, resulted in more pronounced responses compared with the control treatment during the experiment (Van den Brink and Braak, 1999). Statistical differences among the control and restoration treatments at the beginning and the end of the experiment were compared using Kruskal-Wallis test. If a significant difference was found, *post hoc* comparisons among treatments were performed using Wilcoxon test.

Subsequently, we investigated the effects of bivalve addition (bivalve), macrophyte addition (macrophyte), and their interaction (bivalve + macrophyte) on phytoplankton biomass (chlorophyll *a* concentration). For chlorophyll *a* concentrations collected multiple times (i.e., on day 0, 4, 8, 12, 16, 20, 24, 28, and 32), we performed a two-way repeated measures ANOVA (RM-ANOVA) using a restricted maximum likelihood (REML) method. If there was a main effect of bivalve, macrophyte, or their interaction, we performed post-hoc analyses on the data under each treatment. If there was a significant (*p* < 0.05) interaction with time, we performed post-hoc analyses on the data within each sampling time.

The shifts in phytoplankton assemblage composition over time and across treatments were evaluated using a multivariate ordination technique: principal coordinate analyses (PCoA). The PCoA was performed using Hellinger-transformed species data (Legendre and Gallagher, 2001) and a Bray-Curtis dissimilarity matrix. The PCoA was paired with a permutational multivariate analysis of variance (PERMANOVA; Anderson, 2001; Oksanen et al., 2020) to test for statistically significant differences in phytoplankton assemblage composition in different treatments with an *F*-type test (999 permutations) using the same dissimilarity matrix (Bray-Curtis) and transformed species data.

The effect of the addition of filter-feeding bivalves and SMs on the biomass of the cyanobacteria genera was tested using a generalised linear mixed-effects model (GLMM; Bolker et al., 2009; Harrison et al., 2018) with a normal distribution. In separate analyses, dependent variable were (i) total biomass of cyanobacteria, (ii) biomass of filamentous cyanobacteria, and (iii) biomass colonial cyanobacteria. Models were fitted using bivalve, macrophyte and their interaction as fixed effects. All models included mesocosm identity as a random effect. We reported the GLMM marginal *R^2^* (*R2 m*) that describes the variance explained by the fixed effects alone, and the conditional *R^2^* (*R2 C*) that describes the variance explained by both fixed and random effects (Nakagawa and Schielzeth, 2013).

Statistical analyses were performed using R statistical (version 4.0.3) software (R Core Team, 2020). The PRC, PCoA and PERMANOVA were performed using the *vegan* package version 2.5-7 (Oksanen et al., 2020). The RM-ANOVA was performed using the *ez* package version 4.4-0 (Lawrence, 2016). We conducted GLMM using the *glmmTMB* package version 1.1.3 (Brooks et al., 2017). The *MuMIn* package version 1.46.0 (Bartoń, 2022) was used to generate the *R^2^* value of each model.

## 3 Results

### 3.1 Treatment effects on physicochemical parameters

**Figure 2:**
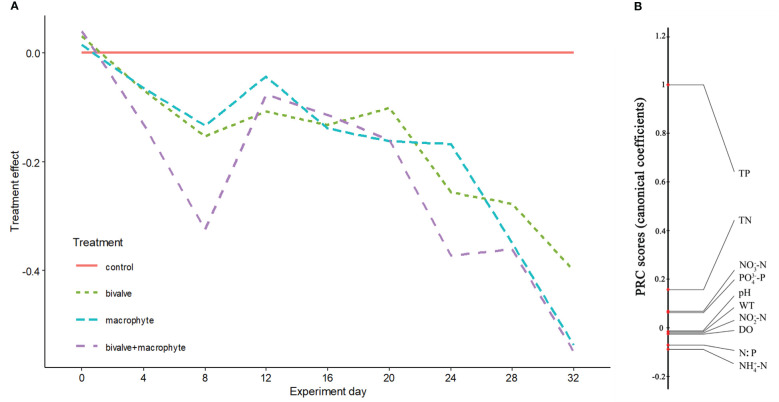
Principal response curves resulting from the analysis of water physicochemical variables. Panel **(A)** represents overall deviation from the control treatment mesocosms (control), for the other restoration treatment mesocosms (bivalve, macrophyte, and bivalve + macrophyte). This is expressed as a canonical coefficient of the first principal component axis (PC1), in comparison with the reference control mesocosms, represented by the zero line. Panel **(B)** shows canonical coefficients for the water physicochemical variables interpreted.

No significant differences were found for the physicochemical parameters among the treatments at the beginning of the experiment (Kruskal-Wallis test: *P* > 0.05; **supplementary Table S1**, **Figure S1**). At the end of the experiment, the biomass of *C. plicata* and the total wet weight of macrophyte increased to 450.2 ± 12.8 standard error (*S.E.*) g/m^2^ and 3257.6 ± 52.9 *S.E.* g L^-1^, respectively. During the experiment, nutrient concentrations in the water shifted in parallel in the restoration treatments (i.e., bivalve, macrophyte and bivalve + macrophyte treatments) relative to the control treatment, with the strongest treatment effects apparent in the bivalve + macrophyte treatment (**Figure 2A**). The principal response curves (PRC) revealed that 39.2% of the total variance present in water nutrient concentrations is explained by treatment (Monte Carlo, *P* < 0.001). Nitrogen and phosphorus loading led to increased nutrient concentrations in the control treatment, but the decline in the nitrogen to phosphorus ratio (N: P) of restoration treatments. Total phosphorus, total nitrogen, phosphate and nitrate nitrogen had high positive scores (**Figure 2B**), with the diagram indicating a decrease with the restoration treatment mesocosms. N: P, ammonia nitrogen and nitrite nitrogen had negative scores (**Figure 2B**), meaning treatment-related increases. At the end of the experiment, addition of filter-feeding bivalves and restoration of submerged macrophyte significantly decreased the concentrations of total phosphorus and total nitrogen (**supplementary Table S1**).

### 3.2 Treatment effects on total phytoplankton

**Figure 3:**
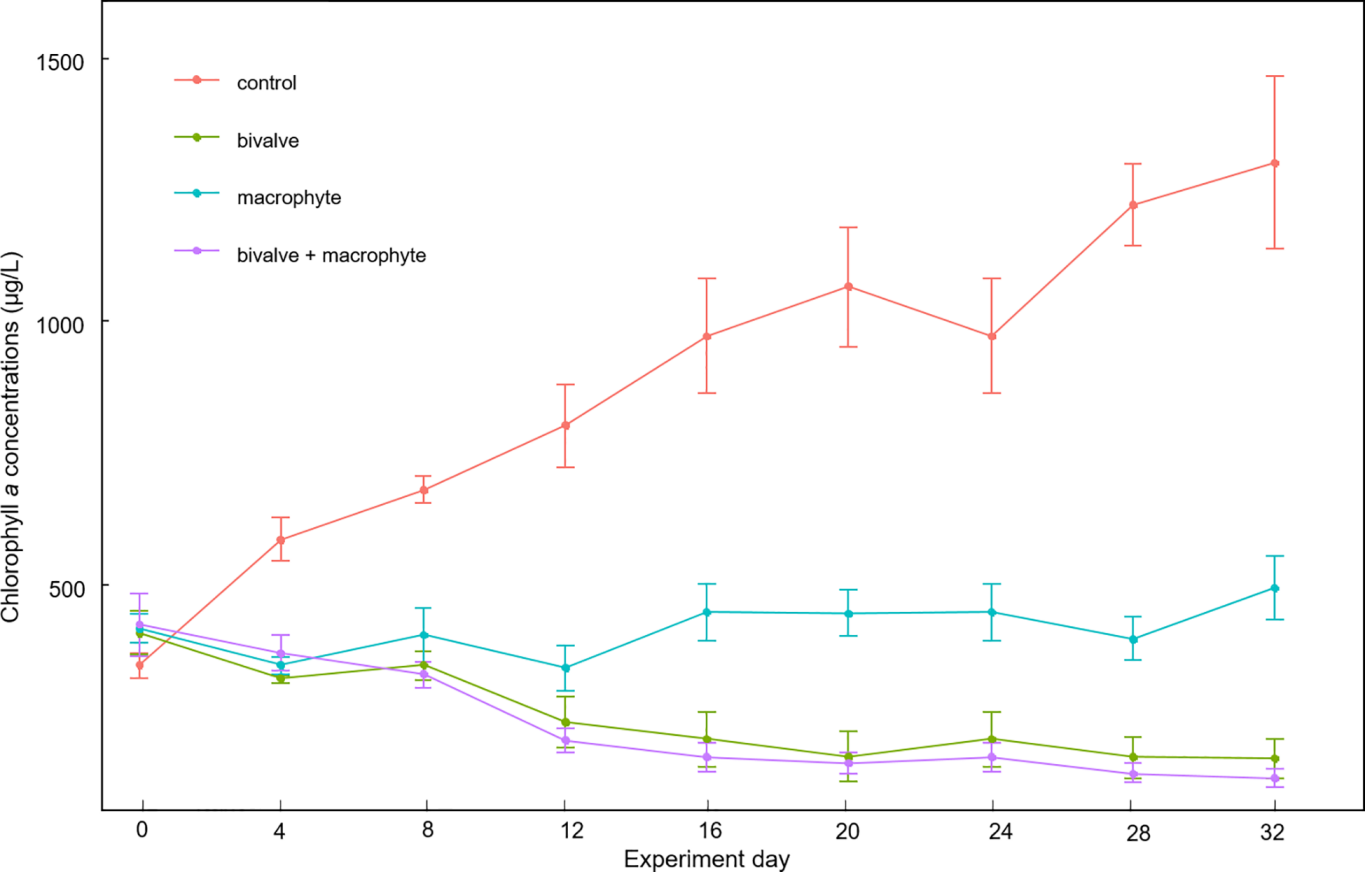
Mean values (± standard error) of temporal variations of chlorophyll *a* concentrations throughout the experiment for the different treatments.

The two-way repeated measures ANOVA results revealed that bivalves (*F*_1, 3_ = 118.6, *p* < 0.001), macrophytes (*F*_1, 3_ = 39.7, *p* = 0.008), and their interactions (*F*_1, 3_ = 31.2, *p* = 0.011) had significant effects on phytoplankton biomass. We found a significant decline in chlorophyll *a* concentrations in the bivalve treatment after day 12 (*p* < 0.05), in addition, a significant decline in the bivalve + macrophyte treatment after day 8 (*p* < 0.05). Chlorophyll *a* concentrations markedly increased in the control treatment but decreased in the bivalve, and bivalve + macrophyte treatments (**Figure 3**). Chlorophyll *a* concentrations remained at a relatively stable level in macrophyte treatment (*p* > 0.05). At the end of the experiment, restoration mesocosms contained 63.7-91.8% less phytoplankton than those of the control treatment. Our analysis indicated a time-by-bivalve interaction (*F*_1, 3_ = 12.8, *p* = 0.037). After day 16, chlorophyll *a* concentrations were significantly lower in both bivalve and bivalve + macrophyte treatments than those in macrophyte treatment (**Figure S2**).

**Figure 4:**
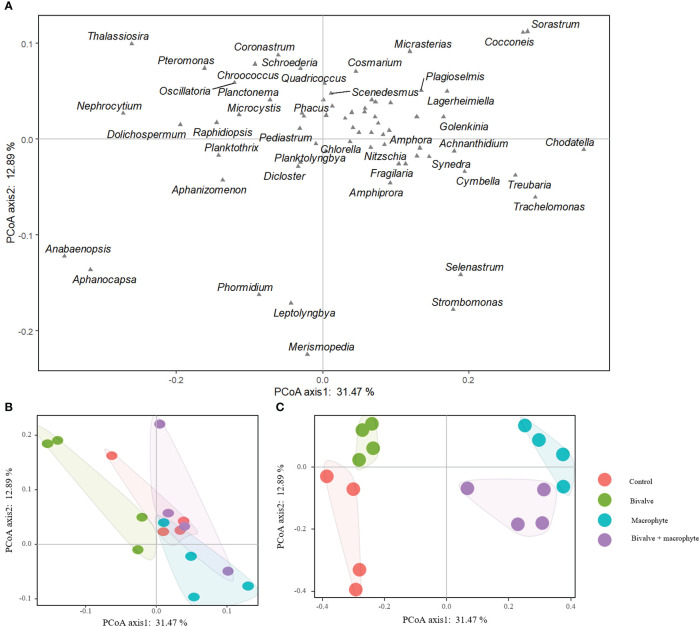
Two-dimensional ordination plots resulting from the principal coordinate analysis on the phytoplankton assemblage composition **(A)** comparing the control and restoration treatments on day 0 **(B)** and day 32 **(C)**.

We found a total of 68 phytoplankton genera throughout the experiment, with representatives from the following classes: Cyanophyceae (14), Bacillariophyceae (14), Chlorophyceae (34), Cryptophyceae (2) and Euglenophyceae (4) (**supplementary Table S2**). The principal coordinate analysis (PCoA) explained 44.36% of the species composition distribution through the first two axes (**Figure 4**). Initially (day 0), no treatments differed significantly in phytoplankton composition (*P* > 0.05; **Table 1**), and chlorophytes dominated the phytoplankton assemblage. By the end of the experiment (day 32), the phytoplankton compositions of the restoration treatments were significantly distinguishable from the controls (**Figure 4**). Addition of bivalves and/or macrophytes induced significant changes in phytoplankton assemblage structure (**Table 1**). Specifically, filamentous cyanobacteria, such as *Anabaenopsis*, *Aphanizomenon*, and *Phormidium*, and colonial cyanobacteria, such as *Aphanocapsa*, became abundant and dominant in the control treatment., while symmetrical desmids (e.g. *Cosmarium*, *Micrasterias*) tended to increase over time in the macrophyte treatment, and the large diatoms (e.g. *Cymbella, Fragilaria, Thalassiosira*) became dominant in the treatments with the addition of filter-feeding bivalves.

**Table 1:** Pseudo *F*-statistics (above diagonal) and *p* values (below diagonal) for pairwise PERMANOVA tests between the control and restoration treatments on two sampling days (day 0 and day 32). .

	Day 0				Day 32			
	Control	Bivalve	Macrophyte	Bivalve + macrophyte	Control	Bivalve	Macrophyte	Bivalve + macrophyte
Day 0-control		1.073	1.614	0.787	5.284	3.793	3.141	3.015
Day 0-bivalve	0.365		1.547	2.297	4.716	3.694	4.568	4.582
Day 0-macrophyte	0.214	0.098		1.228	6.404	6.029	3.763	2.835
Day 0-bivalve + macrophyte	0.683	0.053	0.317		5.463	3.677	2.98	2.225
Day 32-control	0.029	0.037	0.033	0.034		2.952	8.021	6.204
Day 32-bivalve	0.024	0.041	0.027	0.023	0.026		6.926	6.471
Day 32-macrophyte	0.021	0.038	0.026	0.030	0.039	0.033		4.265
Day 32-bivalve + macrophyte	0.027	0.039	0.029	0.029	0.036	0.031	0.033	

### 3.3 Treatment effects on cyanobacteria

By the end of the experiment, the total biomass of cyanobacteria was explained by a positive interaction between bivalve and macrophyte addition. Bivalve and macrophyte addition, as single restoration approaches, resulted in statistically significantly lower cyanobacteria biomass than in the control mesocosms (**Figure S3**). However, in combination, the effects of bivalves and macrophytes partly counterbalanced each other, resulting in a weak antagonistic interaction, where the total biomass of cyanobacteria was higher than the linearly combined (additive) effects of bivalve and macrophyte additions as single restoration techniques. Decreases in filamentous cyanobacteria and colonial cyanobacteria in response to the addition of bivalves and macrophytes as single restoration approaches were similar (**Table 2**). Filamentous cyanobacteria were more sensitive to macrophytes than colonial cyanobacteria, that is, filamentous cyanobacteria biomass decreased more in response to the addition of macrophytes, as single restoration techniques, than the addition of bivalves, while colonial cyanobacteria were more sensitive to bivalves.

**Table 2:** Summary (coefficients and *SE*) of a generalized linear mixed-effects model to explain variations in cyanobacteria taxa biomass as a function of bivalve (presence and absence) and macrophyte (presence and absence).

Biomass (µg/L)	Intercept	Bivalve	Macrophyte	Bivalve ×Macrophyte	*R2 m*	*R2 C*
ln total cyanobacteria	**2.20**	**-1.34**	**-2.08**	**1.04**	0.82	0.87
ln filamentous cyanobacteria	**2.18**	**-1.38**	**-1.92**		0.84	0.88
ln colonial cyanobacteria	**1.65**	**-1.42**	**-1.09**		0.65	0.79

Significant effects (p < 0.05) are highlighted in bold, and nonsignificant effects are left blank.

## 4 Discussion

The development of SMs is considered as an important restoration strategy in eutrophic shallow lakes. Often restoration experiments do not capture the intricacies related to increased nutrient loading and the amount of filter-feeding animals, instead they primarily focus on assessing the effects of SMs (e.g. Bakker et al., 2013). The use of an experimental mesocosm approach is important for investigating the complexity observed in the field and to gain a mechanistic understanding about the single and interactive effects of multiple restoration measures (Amorim and Moura, 2020; Zhang et al., 2021; Boucher-Carrier et al., 2022). During the loading experiments we noticed that restoration of eutrophic waterbodies by manipulation, i.e., addition of filter-feeding bivalves and SMs, had a marked influence on water quality and algal biomass and community composition.

Our results of TP and TN reduction in the restoration treatments demonstrated that the addition of the filter-feeding bivalve *C. plicata* and the recovery of submerged macrophyte can significantly alleviate eutrophication. Although some species of rooted SMs are sensitive to relatively high nutrients and consequently get suppressed under eutrophic conditions (Søndergaard et al., 2010), in our study, the use of *H. verticillata* reduced the nutrient levels. As a rooted submerged macrophyte, *H. verticillata* can obtain nutrients from sediments *via* root uptake and from the water column *via* foliar uptake (Barko, 1982), thus acting as a major nutrient sink. Moreover, filter-feeding bivalves transfer nutrients (especially P) from the water column to the bottom, through excretion as well as biodeposition of faeces and pseudofaeces (Vaughn and Hoellein, 2018).

As expected, changes in nutrient concentrations and stoichiometry, which were influenced by filter-feeding bivalves and SMs, may have altered the phytoplankton assemblage composition during the mesocosm experiment. In agreement, a previous study observed suppressed Cyanobacterial taxa in lakes under P limitation (Havens et al., 2003). At the end of the experiment, the relatively high TN: TP ratios in the macrophyte (average of 37: 1) and the bivalve + macrophyte (average of 42: 1) treatments likely led to the competitive exclusion of cyanobacteria. These results concur with a previous study by Smith (1983) who noticed suppressed cyanobacterial blooms when the TN: TP ratio exceeded 29 to 1. Algae that are incapable of nitrogen fixation are reported to dominate under P-limited conditions (Schindler, 1977; Amano et al., 2010).

High aquatic N: P ratios in lakes are reported in agricultural regions (Arbuckle and Downing, 2001), suggesting that P is the principal production-limiting nutrient. In our study, relatively high TN: TP ratios (≈29) at the beginning indicated a phosphorus-limitation situation. During the loading experiments addition of N and P close to the Redfield ratio (N: P of 16: 1), relieves nutrient limitation, and promotes a much higher phytoplankton biomass development. Phytoplankton biomass showed a gradual increase under nutrient enrichment (the control treatment). Previous studies have reported a linear relationship between chlorophyll *a* concentration and TP for the lower nutrient ranges (TP < 5–100µgL^-1^) and asymptotic behavior at higher ranges (TP >100 µgL^-1^) (Canfield et al., 1984; Phillips et al., 2008; Borics et al., 2013). Noticeably, at the end of the experiment, TP concentration exceeded 100 µgL^-1^, we noticed an increase in phytoplankton biomass with nutrient enrichment, suggesting nutrients control phytoplankton biomass in nutrient‐rich waters (Richardson et al., 2019).

Even in a nutrient-enrichment scenario, as expected, responses to the addition of filter-feeding bivalves (*Cristaria plicata*) alone contributed to the decline in cyanobacterial and total phytoplankton biomass. The overall decline in cyanobacterial and phytoplankton biomass in these mesocosm can be explained by the direct grazing impacts of the filter-feeding bivalves (Gulati et al., 2008). The direct effects of grazing by *C. plicata* led to statistically significant decrease in filamentous or colonial taxa such as the genera *Dolichospermum* (formerly *Anabaena*), *Microcystis*, and *Planktothrix* (Bastviken et al., 1998; Dionisio Pires et al., 2005) which usually form harmful algal blooms. These results are in line with previous studies indicating that cyanobacteria are directly grazed by filter-feeding bivalves (Hwang et al., 2004; Gao et al., 2017), and support the possibility that addition of filter-feeding bivalves will alleviate algal blooms that are associated with eutrophication. Further, several studies have also shown that filter-feeding bivalves are selective feeders and the filtering rate can vary depending on food particle size and bivalve species. Some functional traits of cyanobacteria, including cell size, life form (e.g. single celled, colonial, or filamentous), nutritional deficiency, and toxin production, can prevent them from being grazed by mussels (White and Sarnelle, 2014; Boegehold et al., 2019). For example, *Corbicula fluminea* selectively filtered particles in the range of 0.2–2 µm (Rong et al., 2021), *Dreissena Polymorpha* preferred food particles from 5 to 40 μm (Sprung & Rose, 1988), and *Venerupis corrugatus* filtered out particles of 5 to 13 μm (Stenton-Dozey and Brown, 1992). Although we did not consider selective grazing effects of *C. plicata* on phytoplankton the composition of phytoplankton assemblages significantly differed between the control and the bivalve treatments at the end of the experiment. This result indicates that once nutrient limitation is alleviated, selective grazing would likely be the main factor affecting the structure of the phytoplankton assemblage. Additionally, the phytoplankton assemblages in the filter-feeding bivalve addition treatments appear to have adaptive responses to selective grazing pressure by *C. plicata*, as larger diatoms (e.g. *Cymbella*, *Fragilaria*, *Thalassiosira*) dominated the phytoplankton assemblages.

SMs can also suppress algal growth *via* allelopathic controls and nutrient competition (van Donk and van de Bund, 2002; Mohamed, 2017; Zhu et al., 2021). However, in our experiments, at the end of the experiment a slight, but not significant increase in overall phytoplankton assemblage biomass (chlorophyll *a*) was noticed in the Macrophyte treatment. The phytoplankton blooms occurred when SMs were absent over the course of the nutrient loading experiment (the control treatment). While phytoplankton biomass initially decreased from day 0 to day 4, it then began to slightly increase until day 32, suggesting that SMs did cause a reduction in phytoplankton biomass. The submerged macrophyte *H. verticillate*, has been reported to produce and release allelochemicals that has inhibitory effects on *Chlorella* cell membrane (Zhang et al., 2012) and cyanobacteria (Wang et al., 2006; Gao et al., 2011). Over 32 days, SMs suppressed overall cyanobacteria biomass (**Table 2**), concurring with findings from other experimental studies (Lürling et al., 2006; Barrow et al., 2019; Amorim and Moura, 2020). Further, factors such as light and nutrient competition likely interacted with allelopathic controls in the mesocosms and led to the competitive exclusion of cyanobacteria.

Although unexpected, we found that filter-feeding bivalves in combination with SMs reduced the biomass of cyanobacteria, and noticeably the effect size of this interaction was less than the sum of their individual effects (i.e., an antagonistic interaction). This result is in line with the widely observed antagonistic interactions in freshwater ecosystems (Jackson et al., 2016; Segurado et al., 2018; He et al., 2021), such as nutrient-pesticide effects on benthic invertebrate richness (Chará-Serna et al., 2019), and fish-shrimp effects on zooplankton biomass (He et al., 2021). The mechanism for the antagonistic interactions is largely unknown, however, a possible explanation could be the asymmetry of mean effect size. In our study, the larger magnitude of the submerged macrophyte restoration may have overridden the effect of stock filter-feeding bivalves (**Table 2**), thereby negating its contribution to their net impact on the overall biomass of cyanobacteria (Sala et al., 2000; Barrow et al., 2019). An antagonism between filter-feeding bivalves and SMs was only detected for total cyanobacteria. For filamentous or colonial taxa, there was no significant interactive effects; rather filamentous cyanobacteria were more sensitive to macrophytes and colonial cyanobacteria to bivalves. The differential sensitivity of cyanobacterial taxa to different biomanipulation approaches has been noticed previously (Gazulha et al., 2012; Amorim and Moura, 2020), the reason being that cyanobacteria are a diverse and morphologically complex group of prokaryotes with different key ecological traits thus eliciting disparate responses (Mantzouki et al., 2016; Rangel et al., 2020).

## 5 Conclusions

Our first hypothesis that manipulation *via* addition of filter-feeding bivalves and restoration of submerged macrophyte will likely affect phytoplankton assemblages was confirmed, as this manipulation efficiently decreased water nutrient concentrations and the overall phytoplankton biomass. Since, phytoplankton were dominated by symmetrical desmids (e.g., *Cosmarium*, *Micrasterias*) and the large diatoms (e.g., *Cymbella*, *Fragilaria*, *Thalassiosira*) in the restoration treatments, with the competitive exclusion of cyanobacteria, our results also supported that manipulation could control the growth of cyanobacteria. Contrary to the second hypothesis, an antagonism between filter-feeding bivalves and SMs was detected but only for total cyanobacteria, demonstrating that the larger magnitude of the submerged macrophyte restoration may override the effect of stock filter-feeding bivalves. However, we should also noticed that the addition of bivalves combined with SMs was more efficient at decreasing nutrient concentrations than the isolated addition of bivalves, and at controlling total algal biomass than the isolated restoration of SMs. Overall, our results suggest that manipulation, through introduction of the stock of bivalves as grazers, associated with the restoration of SMs, is an efficient approach for reducing cyanobacterial blooms and alleviating eutrophication.

## Data availability statement

The original contributions presented in the study are included in the article/**Supplementary Material**. Further inquiries can be directed to the corresponding author.

## Author contributions

XD: Conceptualization, Methodology, Investigation, Formal analysis, Writing – original draft, Writing – review & editing, Funding acquisition. DS: Conceptualization, Investigation, Formal analysis, Writing – original draft, Writing – review & editing. HW: Investigation. JY: Resources. HL: Writing – original draft, Investigation. TH: Funding acquisition. All authors contributed to the article and approved the submitted version.
