# Supplementary material for: The combined effects of filter-feeding bivalves (Cristaria plicata) and submerged macrophytes (Hydrilla verticillate) on phytoplankton assemblages in nutrient-enriched freshwater mesocosms
Source: Front Plant Sci. 2023 Jan 23;14:1069593. doi: 10.3389/fpls.2023.1069593 (PMC9899906; doi:10.3389/fpls.2023.1069593)
Supplement: Supplementary file 1 [file DataSheet_1.docx]

Supplementary Material

# Supplementary Figures and Tables

Supplementary Figure and Table legends

**Figure S1** Time series of water temperature (A), pH (B), dissolve oxygen (C), N: P ratio (D), total phosphorus (E), total nitrogen (F), phosphate (G), ammonia nitrogen (H), nitrate nitrogen (I), and nitrite nitrogen (J) across 32-day experiment under different treatments. Shaded areas represent ± *S.E.* across the replicates in each treatment.

**Figure S2** Chlorophyll a concentrations (µg/L) for the different treatments on day 0 (A), 4 (B), 8 (C), 12 (D), 16 (E), 20 (F), 24 (G), 28(H), and 32 (I). A bracket on top connecting the two treatment is annotated with the level of significance.

**Figure S3** Total cyanobacteria (A), filamentous cyanobacteria (B) and colonial cyanobacteria (C) for the different treatments at the end of the experiment. A bracket on top connecting the two treatment is annotated with the level of significance.

**Table S1** Test of differences in physicochemical variables among treatment at the end of the experiment. Results based on Wilcoxon test.

**Table S2** List of phytoplankton species recorded from the control and restoration treatments on two sampling days (day 0 and day 32)


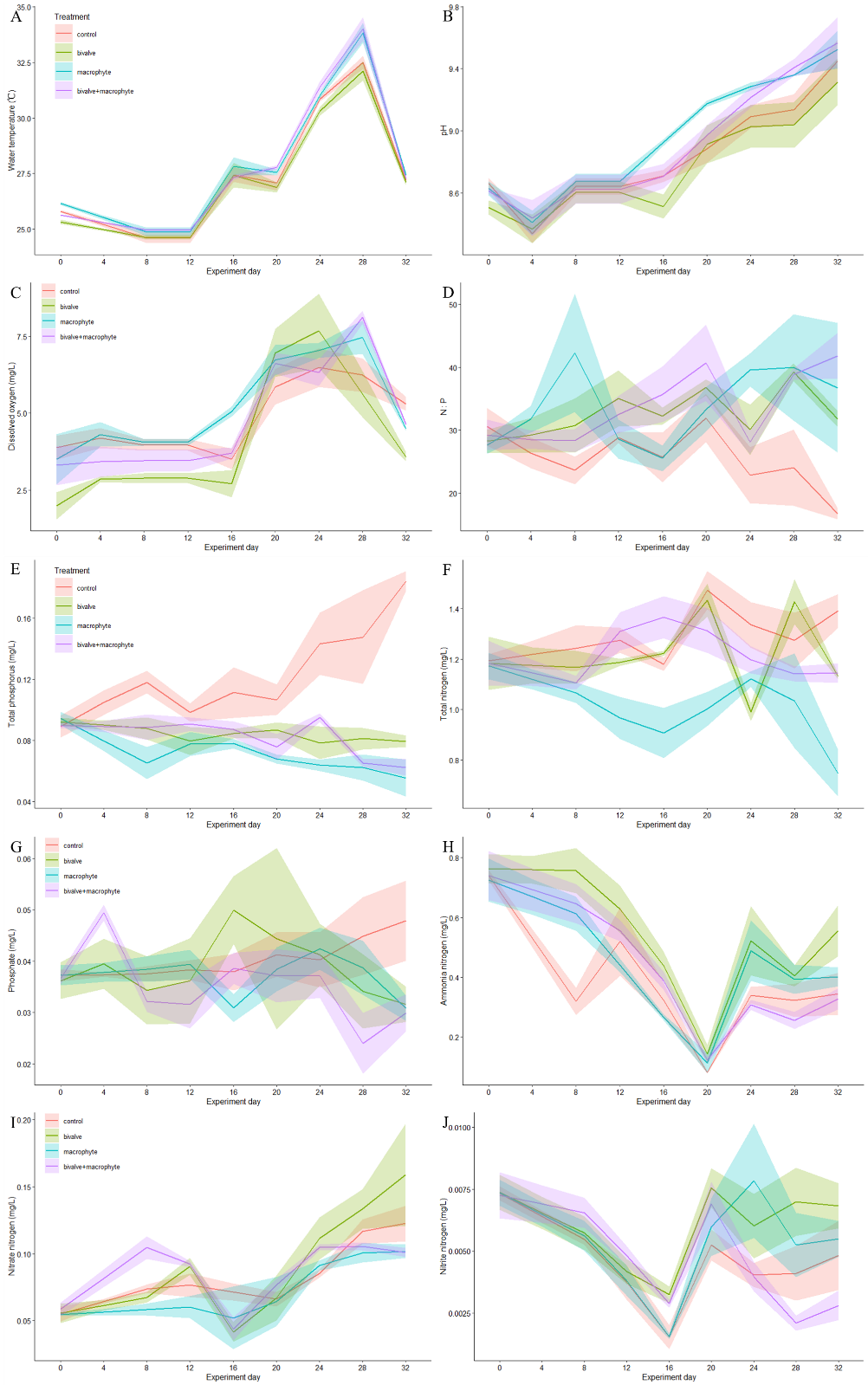


**Figure S1** Time series of water temperature (A), pH (B), dissolve oxygen (C), N: P ratio (D), total phosphorus (E), total nitrogen (F), phosphate (G), ammonia nitrogen (H), nitrate nitrogen (I), and nitrite nitrogen (J) across 32-day experiment under different treatments. Shaded areas represent ± *S.E.* across the replicates in each treatment.


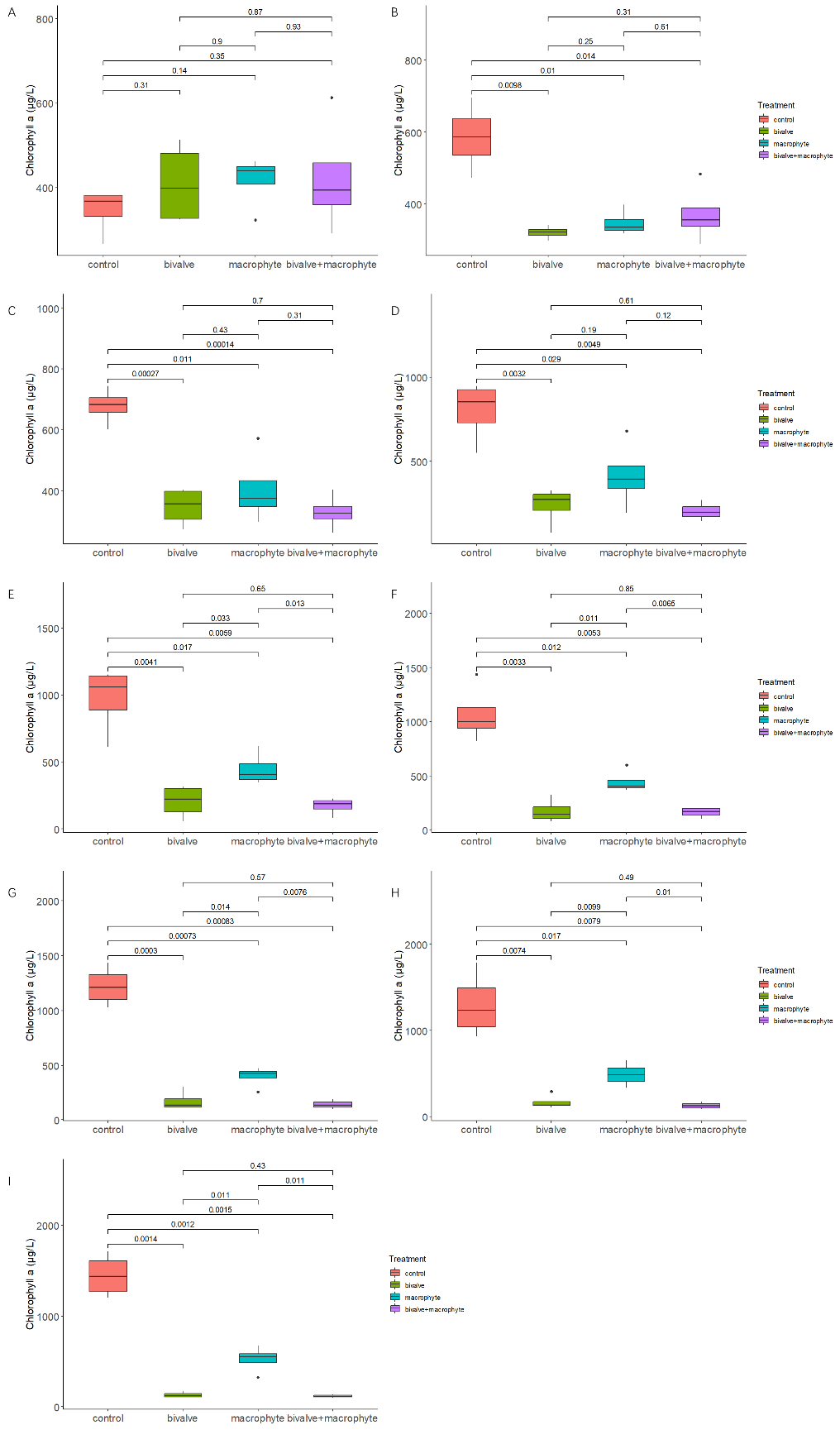


**Figure S2** Chlorophyll *a* concentrations (µg/L) for the different treatments on day 0 (A), 4 (B), 8 (C), 12 (D), 16 (E), 20 (F), 24 (G), 28(H), and 32 (I). A bracket on top connecting the two treatment is annotated with the level of significance.


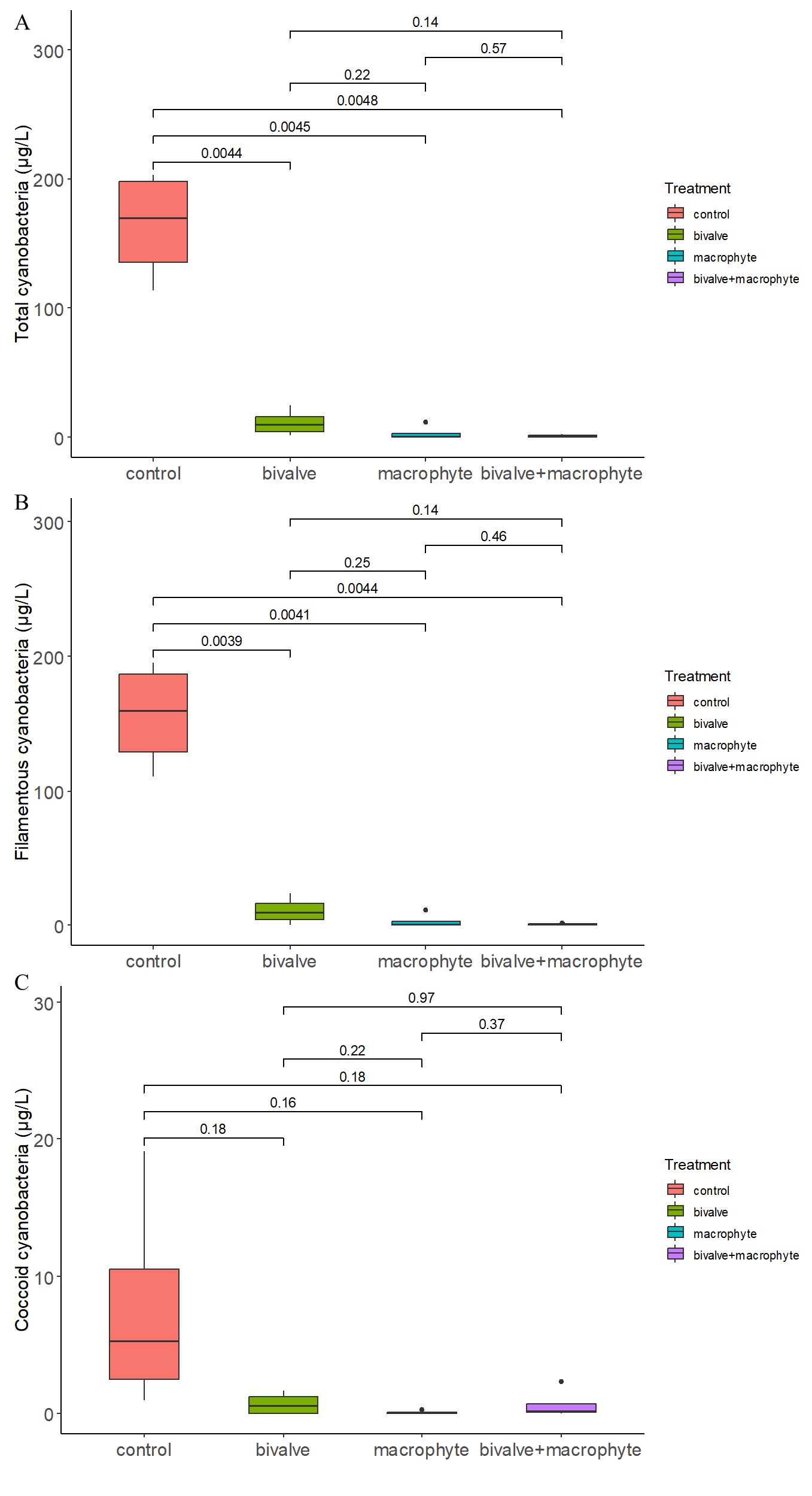


**Figure S3** Total cyanobacteria (A), filamentous cyanobacteria (B) and colonial cyanobacteria (C) for the different treatments at the end of the experiment. A bracket on top connecting the two treatment is annotated with the level of significance.

**Table S1** Test of differences in physicochemical variables among treatment at the end of the experiment. Results based on Wilcoxon test.

|  |  | Control | Bivalve | Macrophyte |
| --- | --- | --- | --- | --- |
| TP | Bivalve | **<0.001** |  |  |
|  | Macrophyte | **0.001** | 0.18 |  |
|  | Bivalve+macrophyte | **<0.001** | **0.049** | 0.66 |
| TN | Bivalve | **0.042** |  |  |
|  | Macrophyte | **0.0036** | **0.036** |  |
|  | Bivalve+macrophyte | **0.042** | 0.78 | **0.026** |
| N: P | Bivalve | **<0.001** |  |  |
|  | Macrophyte | 0.19 | 0.71 |  |
|  | Bivalve+macrophyte | **<0.0073** | **0.091** | 0.71 |
| DO | Bivalve | **0.036** |  |  |
|  | Macrophyte | **0.013** | **0.001** |  |
|  | Bivalve+macrophyte | 0.81 | **0.046** | **0.019** |

**Table S2** List of phytoplankton species recorded from the control and restoration treatments on two sampling days (day 0 and day 32)

| Genera | Day 0 | | | | Day 32 | | | |
| --- | --- | --- | --- | --- | --- | --- | --- | --- |
|  | Control | Bivalve | Macrophyte | Bivalve + macrophyte | Control | Bivalve | Macrophyte | Bivalve + macrophyte |
| *Anabaenopsis* |  |  |  |  | + |  |  |  |
| *Aphanizomenon* | + | + | + | + | + | + |  |  |
| *Aphanocapsa* |  |  |  |  | + |  |  |  |
| *Chroococcus* | + | + | + |  | + | + |  |  |
| *Dactylococcopsis* | + | + | + | + | + | + | + | + |
| *Dolichospermum* | + | + | + | + | + | + |  |  |
| *Leptolyngbya* | + | + | + |  | + |  |  | + |
| *Merismopedia* |  |  |  |  | + | + | + | + |
| *Microcystis* | + | + | + |  | + | + |  |  |
| *Oscillatoria* | + | + | + |  | + | + |  |  |
| *Planktolyngbya* | + | + | + | + | + | + |  | + |
| *Phormidium* | + | + | + |  | + |  |  |  |
| *Planktothrix* | + | + | + | + | + | + |  |  |
| *Raphidiopsis* | + | + | + | + | + | + |  | + |
| *Achnanthidium* | + | + | + | + | + |  | + | + |
| *Amphiprora* | + | + | + | + |  |  |  | + |
| *Amphora* | + | + | + | + | + | + | + | + |
| *Cocconeis* |  |  |  |  |  |  | + |  |
| *Cyclotella* | + | + | + | + | + | + | + | + |
| *Cymbella* | + | + | + | + | + |  | + | + |
| *Diatoma* | + | + | + | + |  |  |  |  |
| *Diploneis* |  |  |  |  |  | + | + |  |
| *Fragilaria* | + | + | + | + | + | + | + | + |
| *Gyrosigma* | + | + | + | + |  | + | + |  |
| *Melosira* | + | + | + | + | + | + | + | + |
| *Nitzschia* | + | + | + | + | + | + | + | + |
| *Synedra* | + | + | + | + | + | + | + | + |
| *Thalassiosira* |  |  |  |  |  | + |  |  |
| *Acutodesmus* | + | + | + | + | + |  | + | + |
| *Ankistrodesmus* | + | + | + |  | + | + | + | + |
| *Chlamydomonas* | + | + | + | + | + | + | + | + |
| *Chlorella* | + | + | + | + | + | + | + | + |
| *Chodatella* |  |  |  |  |  |  | + | + |
| *Closterium* | + | + | + | + | + | + | + | + |
| *Coelastrum* | + | + | + | + |  | + | + | + |
| *Coronastrum* | + | + | + | + |  | + |  | + |
| *Cosmarium* | + | + | + | + | + | + | + | + |
| *Crucigenia* | + | + | + | + | + | + | + | + |
| *Desmodesmus* | + | + | + | + | + | + | + | + |
| *Dicloster* |  |  |  |  |  | + |  | + |
| *Dictyosphaerium* | + | + | + | + |  | + | + | + |
| *Didymogenes* |  |  |  |  | + | + | + | + |
| *Franceia* |  |  |  |  |  | + | + | + |
| *Golenkinia* |  |  |  |  |  | + | + | + |
| *Kirchneriella* | + | + | + | + | + | + | + | + |
| *Lagerheimiella* | + | + | + | + |  | + | + |  |
| *Micrasterias* |  |  |  |  |  | + | + | + |
| *Nephrocytium* |  |  |  |  | + | + |  |  |
| *Oocystis* | + | + | + | + | + | + | + | + |
| *Pediastrum* | + | + | + | + | + | + | + | + |
| *Planctonema* | + | + | + | + | + | + | + | + |
| *Pteromonas* |  |  |  |  | + | + | + |  |
| *Quadricoccus* | + | + | + | + |  | + | + |  |
| *Quadrigula* |  |  |  |  | + | + | + | + |
| *Scenedesmus* | + | + | + | + | + | + | + | + |
| *Schroederia* | + | + | + | + | + | + | + | + |
| *Selenastrum* |  |  |  |  | + |  | + | + |
| *Sorastrum* |  |  |  |  |  |  | + |  |
| *Spondylosium* | + | + | + | + | + | + |  | + |
| *Tetraedrom* | + | + | + | + | + | + | + | + |
| *Tetrastrum* | + | + | + | + | + | + | + | + |
| *Treubaria* |  |  |  |  |  | + | + | + |
| *Campylomonas* | + | + | + | + | + |  | + | + |
| *Plagioselmis* | + | + | + | + | + |  | + | + |
| *Euglena* | + | + | + | + | + | + | + | + |
| *Phacus* | + | + | + | + | + |  |  |  |
| *Strombomonas* |  |  |  |  | + |  | + | + |
| *Trachelomonas* |  |  |  |  | + | + | + | + |

*Note:* + indicates the presence of phytoplankton species.
